# Cluster‐Randomized Trials in Emergency Care Research

**DOI:** 10.1111/acem.70181

**Published:** 2025-10-28

**Authors:** Howard S. Kim, Jacob M. Schauer, Jody D. Ciolino

**Affiliations:** ^1^ Department of Emergency Medicine Northwestern University Feinberg School of Medicine Chicago Illinois USA; ^2^ Center for Dissemination & Implementation Science Northwestern University Feinberg School of Medicine Chicago Illinois USA; ^3^ Division of Biostatistics, Department of Preventive Medicine Northwestern University Feinberg School of Medicine Chicago Illinois USA

## Abstract

**Objective:**

Cluster‐randomized trials (also called group‐randomized trials) are increasingly common in emergency care research. In such trials, groups of participants are allocated to different interventions based on naturally occurring “clusters,” such as clinics, hospitals, or emergency medical services agencies. In this methodological review, we introduced key terminology and features of cluster‐randomized trials, described common rationales for cluster‐randomization and its most common limitations, and offered brief advice for conducting and critically appraising cluster‐randomized trials in emergency care research.

**Results:**

Researchers elect to use cluster‐randomization when individual participant randomization is not preferred or not possible. Common reasons include a desire to limit contamination between study groups, logistical convenience relating to trial administration or study procedures, or the use of an intervention that is naturally group‐oriented, such as an educational intervention or clinical decision support tool that is directed toward influencing clinician behaviors. Although cluster‐randomization has advantages in these contexts, this approach also comes with some notable weaknesses, such as inflated sample size requirements, greater difficulty in blinding participants and researchers, and an increased risk of baseline imbalances between comparator groups. When reading and critically appraising cluster‐randomized trials, emergency clinicians should consider whether researchers have appropriately justified group over individual randomization, accounted for different levels of clustering and the degree of correlation between participants within clusters (intracluster correlation), and appropriately consented various levels of participants to study participation.

**Conclusions:**

Cluster‐randomized trials are frequently used in emergency care research, especially as researchers are increasingly evaluating educational or electronic health record interventions that are naturally group‐oriented or have a high risk of contamination. After reading this review, emergency medicine clinicians and researchers will have a foundational understanding of key cluster trial features and will be able to assess the quality and limitations of emerging evidence.

## 1 Introduction

Randomized clinical trials are considered to be the most rigorous method for evaluating the efficacy of an intervention. Most commonly in clinical trials, individual participants are randomized to receive one intervention or another. However, a growing number of trials in emergency care research are employing *cluster randomization* to allocate a group of participants to different interventions utilizing naturally existing “clusters” such as clinics, hospitals, or emergency medical services (EMS) agencies. Individual participants enrolled in the trial then follow the randomization assignment of their overarching clustering unit.

Recent cluster‐randomized trials in emergency care research have addressed a number of research questions with direct relevance to the practice of emergency medicine, such as whether normal saline or a balanced crystalloid is the preferred intravenous fluid solution for routine ED use, or the optimal defibrillation strategy for refractory ventricular fibrillation [1, 2]. In order to determine whether to incorporate emerging evidence into their everyday practice, emergency clinicians must be able to critically appraise new literature and assess the quality and limitations of published findings. Although Wears et al.'s seminal review of statistical methods for analyzing clustered data remains relevant over two decades since its publication [3], that advanced statistical review is better oriented towards researchers analyzing clustered data. The purpose of this present review is to orient the general emergency clinician to cluster‐randomized trial designs and provide a basic framework for assessing the quality of cluster trials when determining whether to incorporate new research findings into clinical practice. This review may also be useful to emergency medicine researchers who are planning to conduct a clinical trial and are considering whether to use an individually randomized or cluster‐randomized design.

In this methodological review, we introduce and describe important features of cluster‐randomized trials (also called group‐randomized trials), including key terms, common rationales for employing cluster‐randomization, and specific analysis considerations. Importantly, this work builds upon prior foundational reviews of cluster‐randomized trials [3, 4, 5, 6, 7] and highlights recent examples of cluster‐randomized trials relevant to emergency care (Table 1).

**TABLE 1:** Examples of cluster‐randomized trials in emergency care research.

Study title	Study question	Trial design	Clustering unit, intervention deliverer, inference unit	Rationale for cluster randomization	Study conclusion
1. A multifaceted intervention improves prescribing for acute respiratory infection for adults and children in ED and urgent care settings [8]	Are behavioral interventions more effective than education in reducing unnecessary antibiotic prescribing for acute respiratory infections among ED and urgent care clinicians?	Multicenter Two arm Unblinded Pragmatic Parallel Cluster	5 EDs and 4 UCCs within 3 academic health systems, stratified randomization within each health system Third Party Resource EHR Clinicians working within the 5 EDs and 4 UCCs (clinician‐level prescribing rate)	Limit Contamination	Behavioral interventions were not more effective than education in reducing unnecessary antibiotic prescribing, which decreased among all clinicians in all clusters during the trial
2. Effect of a strategy of initial laryngeal tube insertion vs endotracheal intubation on 72‐hour survival in adults with out‐of‐hospital cardiac arrest (PART) [9]	Is an initial airway management strategy using laryngeal tube insertion compared with endotracheal intubation more effective in achieving survival to 72 h among adults with out‐of‐hospital cardiac arrest?	Multicenter Two arm Unblinded Pragmatic Crossover Cluster	27 EMS agencies randomized in 13 clusters Paramedics within those agencies Individual patients: survival to 72 h	Individual Infeasibility: EMS personnel perceived event‐level (e.g., blinded envelope or telemetry) and alternate‐day randomization as adding unacceptable complexity and delays. Blinded equipment pouches were deemed impractical given the range of different airway equipment currently carried by EMS units.	Laryngeal tube insertion was more effective than endotracheal intubation in reaching survival to 72 h among adults with out‐of‐hospital cardiac arrest
3. Defibrillation strategies for refractory ventricular fibrillation (DOSE VF) [2]	Are double sequential external defibrillation and/or vector‐change defibrillation more effective than standard defibrillation in achieving survival to hospital discharge among adults with shock‐refractory ventricular fibrillation?	Multicenter Three Arm Unblinded Crossover Cluster	6 EMS agencies randomized in 6 clusters Paramedics within those EMS agencies Individual patients: survival to hospital discharge	Limit Contamination	Both double sequential external defibrillation and vector‐change defibrillation were more effective than standard defibrillation in achieving survival to hospital discharge among adults with shock‐refractory ventricular fibrillation
4. Palliative care initiated in the emergency department (PRIM‐ER) [10]	What is the effect of an ED‐based multi‐component palliative care intervention on reducing hospital admission in older adults with serious illness?	Multicenter Two Arm Unblinded Pragmatic Stepped Wedge Cluster	29 EDs randomized in 29 clusters Third Party Resource EHR Individual ED visits: hospital admission	Group intervention: educational interventions are best delivered at a group level Logistical Convenience: human resources required to deliver the small group, simulation‐based communication skills training	Implementing a multi‐component intervention to initiate palliative care in the ED did not reduce hospital admissions among seriously ill older adults.
5. Does COVID‐19 vaccine education messaging increase vaccine acceptance? (PROCOVAXED) [11]	Does provision of COVID‐19 vaccine educational messaging increase vaccine acceptance and uptake in unvaccinated ED patients?	Multicenter Two Arm Unblinded Crossover Cluster	30 one‐week blocks of time within each of 7 EDs, thus resulting in the randomization of 210 clusters Third party resource Clinicians Individual patients: COVID‐19 vaccination acceptance and receipt	Individual Infeasibility: limits on research personnel in patient care areas during the COVID‐19 pandemic that precluded 24/7 study enrollment and necessitated randomizing by week	Providing multi‐component COVID‐19 vaccine education increased vaccine acceptance and uptake among unvaccinated ED patients.
6. User centered clinical decision support to implement initiation of buprenorphine for opioid use disorder in the emergency department (EMBED) [12]	Does a user‐centered clinical decision support tool improve rates of buprenorphine initiation among ED patients with opioid use disorder?	Multicenter Two Arm Pragmatic Parallel Cluster	21 EDs in 18 clusters EHR Individual patients: initiation of ED buprenorphine	Limit Contamination Interest in implementation outcomes at the cluster level	A user‐centered clinical decision support tool did not improve rates of buprenorphine initiation among ED patients with opioid use disorder
7. A multifaceted intervention to improve patient knowledge and safe use of opioids (EMC^2^) [13]	Which strategy is more effective for improving ED patients' ability to safely dose their prescribed opioid medications: EHR‐based discharge education, HER based discharge education+daily text messages, or usual care? care intervention on reducing hospital admission in older adults with serious illness?	Single Center Three‐Arm Pragmatic Cluster	116 ED physicians in a single ED randomized in 116 clusters EHR Individual patients: Safe Opioid Use Knowledge	Group intervention: EHR interventions are best delivered at a group level	An EHR‐based discharge education intervention improved safe use of opioids among ED patients compared to usual care.
8a. Balanced crystalloids versus saline in critically ill adults (SMART) [14] AND 8b. Balanced crystalloids versus saline in non‐critically ill adults (SALT‐ED) [1]	Dose the use of balanced crystalloid fluids versus normal saline in critically ill adults result in lower rates of major adverse kidney events within 30 days Dose the use of balanced crystalloid fluids versus normal saline in non‐critically ill adults reduce the number of hospital free days within 30 days?	Single center Two arm Unblinded Pragmatic Crossover Cluster Single center Two arm Unblinded Pragmatic Mult Crossover Cluster	5 Intensive care units within a single hospital randomized in 2 clusters (one group of 3 ICUs that admit patients from the ED, and one group of 2 ICUs that admit patients from the operating room) No intervention deliverer Individual patients: major adverse kidney events within 30 days A single hospital ED; each month of the 16 month trial was treated as a cluster, with crossover to the other study arm in each subsequent arm; the first month of the sequence was randomly assigned. No intervention deliverer Individual patients: number of hospital free days within 30 days	Group intervention: EHR interventions are best delivered at a group level Limit Contamination Group intervention: EHR interventions are best delivered at a group level Limit Contamination	Among critically ill adults, the use of balanced crystalloids compared to normal saline resulted in a lower rate of major adverse kidney events. Among non‐critically ill adults, the use of balanced crystalloids compared to normal saline did not reduce the number of hospital‐free days.
9. Implementation of evidence‐based practice for benign paroxysmal positional vertigo in the ED (DIZZTINCT) [15]	Does an educational intervention and clinical decision aid increase use of evidence‐based treatment of benign paroxysmal positional vertigo?	Multi Center Two Arm Unblinded Stepped Wedge Cluster	6 EDs randomized as 6 clusters; as this was a stepped wedge trial the order of rollout was randomized Third party resource Individual ED visits: documentation of evidence‐based treatment	Group intervention: educational interventions are best delivered at a group level	Implementation of an educational intervention and clinical decision aid increased documentation of evidence‐based treatments for BPPV
10. Decision support intervention and anticoagulation for emergency department atrial fibrillation (O'CAFÉ) [16]	Does physician education and clinical decision support improve initiation of anticoagulation among ED patients with atrial fibrillation?	Multicenter Two arm Unblinded Single crossover Pragmatic Stepped wedge Cluster	13 EDs randomized as 9 clusters Third party resource EHR Individual patients: initiation of ED anticoagulation	Group intervention: educational interventions and EHR interventions are best delivered at a group level	Education and clinical decision support did not improve initiation of anticoagulation among ED patients with atrial fibrillation

## 2 Key Concepts and Terminology

One of the earliest examples of cluster‐randomization is in the evaluation of school‐based interventions [17, 18, 19], which provide a convenient illustration of the concept of clustering or grouping. Imagine that an educational researcher wishes to evaluate a novel method of teaching high school students. Although the new teaching method aims to improve knowledge among individual students, randomizing individual students to different teaching methods might be problematic from both a logistical and analytical perspective. From a logistical perspective, students are naturally grouped into classrooms, so it is difficult for a single teacher to administer two different teaching methods to individual students in the same classroom. Thus, a randomized trial of an educational intervention might elect to randomize classrooms to different teaching methods rather than individual students. In the same way, a clinical trial evaluating a new teaching method for resident physicians might elect to randomize residency programs rather than individual residents [20], or alternatively, a clinical trial that aims to educate ED physicians about a new skill or resource might elect to randomize EDs rather than individual ED physicians [10, 15, 21, 22].

From an analytical perspective, the fact that students are naturally clustered within classrooms introduces the important statistical concept of *intra‐cluster correlation*. The basic premise is that individuals who exist in naturally occurring clusters tend to be more similar to each other than they are to individuals from other clusters; in other words, there is natural within‐cluster correlation and between‐cluster variation. We might expect, for example, that EM residents (i.e., individuals) within a specific residency program (i.e., clusters) are more similar to one another than they are to residents from other programs based on the particular recruitment priorities and unique residency cultures of each program [23]. By the same token, patients at a county hospital ED in Denver are more likely to be similar to each other than they are to patients at an academic tertiary referral hospital ED in Baltimore [24, 25]. Statistically, the concept of intra‐cluster correlation is represented by the intra‐cluster correlation (ICC) coefficient, which ranges from 0.0 to 1.0, with higher values indicating stronger correlation within clusters. Researchers use the ICC coefficient to calculate sample‐size requirements when designing a cluster‐randomized trial and to analyze clustered data after trial data collection is complete.

Importantly, intra‐cluster correlation should be accounted for even if the study is not a cluster‐randomized trial (i.e., it is an observational study or an individually randomized trial) [24, 26, 27, 28, 29, 30]. Failure to account for similarity within naturally occurring clusters can lead to inaccurate and misleading statistical inferences, which we describe in more detail below in section 5.2. Hence, while clustering has a specific logistical role as the unit of randomization (and in many cases, method of intervention delivery) in cluster‐randomized trials, the concept of intra‐cluster correlation also plays an important role when analyzing data from observational studies with naturally clustered data, such as a retrospective cohort study of assessment scores among U.S. emergency medicine residents [23] or a multi‐center non‐randomized trial of protocolized steroids for pediatric asthma among Florida EMS agencies [31], and when analyzing naturally clustered data from a trial that randomizes individuals rather than clusters [24, 25].

Finally, there are a number of additional terms that are often combined with cluster‐randomization to describe specific clinical trial design features, such as a “pragmatic cluster‐randomized crossover trial” or a “stepped‐wedge cluster‐randomized hybrid effectiveness‐implementation trial.” We note that each of these terms (pragmatic, crossover, stepped‐wedge, hybrid effectiveness‐implementation, sequential multiple assignment, etc.) refers to additional trial features outside of the decision to randomize by individuals or clusters [32, 33]. The term *cluster‐randomization* refers specifically to the selection of a group or cluster as the randomization unit rather than individual participants, which is not altered by the addition or specification of any additional trial design features. We describe a selection of additional trial design features often paired with cluster trials in Box 1, the most common of which is a pragmatic trial due to the tendency to roll out an intervention by clusters rather than individual patients in real‐world conditions. However, it is important to note that although many pragmatic trials take place within settings that have naturally occurring clusters [12, 24], not all pragmatic trials are cluster‐randomized and not all cluster‐randomized trials are “pragmatic.”

BOX 1 **Additional trial design features often paired with cluster‐randomized trials.**

Numerous additional terms are often combined with the term “cluster‐randomization” to describe the complete design features of a clinical trial, such as a “pragmatic cluster‐randomized crossover trial.”

*Pragmatic* trials are designed to closely approximate real‐world conditions by enrolling a broadly representative patient population, using existing infrastructure to streamline trial execution and data collection procedures, and measuring a broad range of clinically meaningful outcomes.

*Hybrid effectiveness‐implementation* trials focus on both implementation outcomes (e.g., adoption, reach, fidelity) and effectiveness outcomes (e.g., survival, cure rate) after an intervention has been previously shown to be efficacious in tightly controlled smaller trials.

*Crossover trials* assign participants to cross over from treatment to control conditions (and/or vice‐versa) in a uni‐directional or bi‐directional fashion to contribute observations to both conditions.

*Stepped‐wedge* trials are a specific subset of cluster trials that involve the sequential rollout of an intervention across all participating sites over discrete time blocks, wherein sites are randomized to implement the intervention at a given time (i.e., a “step”) during the trial.

## 3 Common Rationales for Selecting Cluster‐Randomization Over Individual Randomization

Researchers may choose to randomize at the cluster level rather than the individual level for a number of reasons [5, 34]. First, some interventions are naturally delivered at a group level, such as educational interventions for clinicians or added‐resource interventions for entire EDs. Common examples of group interventions include clinical decision support (CDS) tools [12, 13, 16, 35], which are typically launched through a hospital‐wide update of the electronic medical record system, educational initiatives, which are most efficiently delivered to large groups rather than individual clinicians, or newly added ED resources [10, 15, 21], such as the offering of a peer recovery specialist for patients with substance use disorder. These group‐based interventions also tend to evaluate outcomes of interest that are best characterized at a group level, such as the proportion of ED visits receiving a high‐value care metric, such as buprenorphine prescribing for opioid use disorder or anticoagulation prescribing for atrial fibrillation [12, 16, 21].

Second, the desire to avoid contamination between comparator groups can often drive the decision to cluster‐randomize [1, 2, 8, 12, 14]. Although trials that evaluate *added‐resource interventions* could randomize individual patients to receive that resource, clinicians participating in the trial might eventually override a patient's randomization to a control condition based on their knowledge of that resource from prior patients. Conversely, cluster‐randomized trials could also be perceived as a mechanism to maximize adherence to an intervention, as clinicians participating in an individually randomized trial might choose not to deliver an intervention due to a lack of knowledge or familiarity with how it should be delivered.

However, it should be noted that one limitation of emergency medicine cluster trials is that clinicians may practice at more than one site [36]—which could introduce a degree of contamination. In such cases, researchers could assign the clinician to the cluster at which they work the most [8] or exclude those clinicians [12]. Alternatively, a clinical context or target population might be deemed to be too high acuity for the complexities of individual patient randomization—this is often the case for EMS trials in which a difference of seconds can meaningfully change outcomes. For example, a recent trial in Denmark evaluated whether adding video streaming to 911 calls improves the accuracy of dispatching EMS services [37]. Researchers elected to randomize by clusters of EMS dispatchers rather than individual 911 calls because they felt that it would be too difficult for individual dispatchers to toggle between audio‐only vs. video streaming given that 911 calls arrive spontaneously and may require immediate action. Similarly, in a trial evaluating laryngeal tube intubation versus supraglottic airway insertion for out‐of‐hospital cardiac arrest, researchers elected to randomize by clusters of EMS agencies because unmasking the randomization assignment of each individual patient (and carrying two concealed equipment bags) was deemed to be impractical and represent an unnecessary risk of delayed treatment.

Third, trials delivering particularly intensive interventions might cite cluster randomization as a means to facilitate intervention delivery and reduce the overall burden of trial administration [10]. In the Primary Palliative Care for Emergency Medicine (PRIM‐ER) trial, 29 U.S. EDs were randomized to receive a multicomponent intervention to initiate palliative care in patients with serious illness. The intervention was deemed to be too intensive to be delivered at an individual clinician level as it included a multidisciplinary education program, simulation‐based workshops on serious illness communication, an electronic health record CDS tool, and audit and feedback of ED clinician actions [10]. Relatedly, cluster randomization often overcomes logistical challenges posed by “pragmatic” trial designs where an intervention is embedded within usual care and delivered to all recipients in real‐world conditions [38]; such cluster randomized pragmatic trials can produce insights directly relevant to the conditions faced by healthcare systems in the real world [39].

Other reasons for cluster‐randomization include a desire to avoid “disappointment” effects [40], whereby individual participants are “disappointed” when they are allocated to not receive an intervention that they perceive to be beneficial. Cluster‐randomization can thus facilitate recruitment of individual participants by randomizing the site to a condition earlier in the timeline and removing the potential disappointment of real‐time randomization. In other cases, researchers might use cluster‐randomization when they are also interested in observing the indirect effects of an intervention—such as the herd immunity that might result from a vaccine trial or the spillover from an educational intervention in one clinical area to another—or implementation outcomes that are typically assessed at the group level [12]. Additionally, researchers might prefer to use cluster‐randomization when they consider individual randomization methods to be infeasible in the specific clinical context, such as the highly acute and time‐sensitive decision to intubate a patient in the pre‐hospital setting [9], or in atypical research environments, such as an ongoing pandemic where minimizing research staff exposure is a critical concern [11].

**FIGURE 1:**
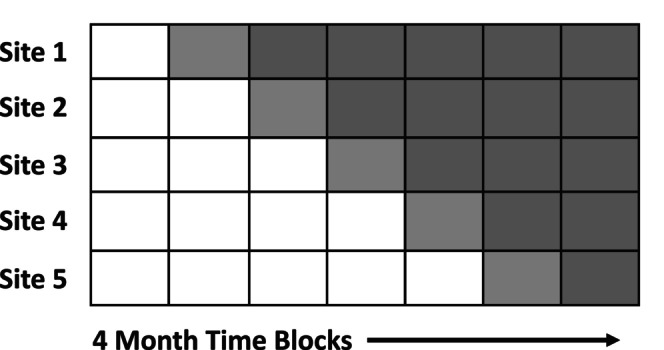
Stepped‐wedge rollout trial diagram. Example of a stepped‐wedge rollout trial in which five sites are randomized to sequentially rollout an intervention over a number of discrete time blocks. Each site contributes study observations in both the control (white) and intervention (shaded) time blocks.

Finally, investigators may employ different cluster‐randomized trial designs, such as a *parallel group design, stepped‐wedge rollout design* [10, 15, 16] or *bi‐directional cross‐over design* [1, 2, 9, 14]. Parallel group designs are the most conventional cluster‐randomized design, in which clusters are randomized at a single common timepoint (or in waves at a series of fixed timepoints) and followed over the same pre‐specified interval. *Stepped‐wedge trials* involve the sequential rollout of an intervention across all participating sites over a number of discrete time blocks, wherein sites are randomized to implement the intervention at a given time (i.e., a “step”) during the trial (Figure 1) [41]. Participating sites contribute data from both their pre‐ and post‐rollout blocks to compare the effect of treatment vs. control; for this reason, stepped‐wedge trials may be better described as pre‐post prospective cohort studies that randomize the sequence of intervention rollout rather than as randomized trials. Though analyses of stepped‐wedge designs tend to be more powerful than analyses of parallel trials, they involve comparisons both between study arms and across time—this puts them at greater risk of bias due to inadequate temporal adjustment, within‐site contamination, and disparate participant selection [42]. However, stepped‐wedge designs can be justified in scenarios where it would be unethical to withhold the intervention from half of participating sites, infeasible to support rollout of a complex intervention across multiple sites simultaneously, or where a stepped‐wedge design offers meaningful increases in power relative to a parallel trial [41, 42, 43, 44, 45]. In bi‐directional *crossover trials*, clustering units cross over from treatment to control conditions (and vice‐versa) to contribute observations to both comparator groups. This can be of benefit when researchers wish to improve a trial's statistical power within a fixed number of participating sites; however, it should be noted that crossover trials are subject to similar bias risks as stepped‐wedge designs [40].

As illustrated in these examples, the rationale for selecting cluster‐randomization often depends on the type of intervention being evaluated and the intended recipient of the intervention. Eldridge et al. previously established a typology of intervention types based on the primary rationale for adopting a clustered design [46], which we have adapted to include more contemporary intervention examples applicable to emergency care research (Table 2).

**TABLE 2:** Types of interventions utilized in cluster‐randomized trials.

Type	Intervention target	Intervention deliverer	Inferential target	Rationale for cluster randomization	Examples
1. Patient facing, clinician delivered	Patients	Clinicians	Patients, clinicians	To decrease contamination and increase adherence. In other words, to ensure that patients randomized to Group A do not receive Treatment B, and that patients randomized to Group B do not receive Treatment A.	In a trial testing the effectiveness of double sequential defibrillation versus vector change defibrillation for patients with refractory ventricular fibrillation [2], paramedic agencies (clustering unit) were randomized to employ one strategy vs. the other. An individually randomized trial might have led paramedics to inadvertently administer one treatment over the other due to confusion over the patient's randomization assignment, particularly in the stress and urgency of a cardiac arrest. Thus, the risk of contamination can be decreased by tasking clinicians with delivering a single consistent intervention rather than delivering different interventions from patient to patient.
2. Patient facing, externally delivered	Patients	Third party resource: educational materials	Patients	To decrease contamination and increase adherence Individual Infeasibility: Not possible to tailor an educational intervention to each patient	In a trial evaluating the efficacy of a COVID‐19 vaccine messaging platform delivered in ED waiting rooms [11], sites were randomized to play a four‐minute video, provide a one‐page informational flyer, or deliver a brief scripted message from an ED physician or nurse. The primary outcome was vaccine acceptance by patient survey and the secondary outcome was vaccine receipt at 30 days. An individually randomized trial was not preferred because patients in the ED waiting room might overhear or have access to other intervention types beyond their individual randomization assignment.
3. Clinician facing, externally delivered	Clinicians	Third party resource: electronic health record, expert facilitators, educational materials, data feedback	Primary: patients Secondary: clinicians	Decrease contamination and increase adherence Group intervention: educational interventions are best delivered at a group level Logistical convenience: expert facilitation requires pairing a limited number of outside experts with time‐ and schedule‐limited clinicians Interest in indirect spillover effects	In a trial evaluating the effect of a clinical decision support tool to implement initiation of buprenorphine for opioid use disorder [12], sites were randomized to receive a clinical decision support tool embedded in the electronic health record to facilitate buprenorphine prescribing. An individually randomized trial (in which each ED visit was randomized to have the CDS tool enabled vs. not) might be at risk for contamination, as an ED clinician would have access to the CDS tool for some patients but not others; some of the prescription facilitation from “treatment” patients might encourage them to prescribe buprenorphine for “control” patients. In this instance, cluster‐randomization could be conducted at multiple levels: the ED clinicians or the site. Selecting ED clinicians as the clustering unit may be difficult logistically as typically CDS tools are rolled out site‐wide rather than to specific users; additionally, clinicians randomized to control might observe their colleagues at the same site using the tool and be influenced.
4. Organization facing, externally delivered	Organizational structure or culture	External force: change in scheduling	Patients, clinicians	Intervention necessity intervention cannot be delivered any other way because it manipulates the physical or social environment	In a trial evaluating a more flexible work hour schedule for surgical residents in training, residency programs were randomized to continue current standard scheduling vs. adopt new “flexible” work scheduling. The trial's primary outcome was patient safety and secondary outcomes included resident physician well‐being and satisfaction. An individually randomized trial was not possible given that all resident physicians within a given program must be on the same type of scheduling policy in order to create a workable schedule [20].

## 4 Common Limitations of Cluster‐Randomized Trials

The potential advantages of cluster‐randomization should be weighed against its potential weaknesses. First, cluster‐randomized trials typically require larger total sample sizes compared to individually randomized trials, or put another way, hypothesis tests involving clustered data tend to have lower power than non‐clustered data. The decrease in power is a function of the correlation within clusters (ICC), or equivalently, greater variation between clusters. When participants in a cluster are more similar to each other (i.e., higher ICC), they provide less information about the broader population of participants. To make up for that, more clusters and participants are typically needed to detect treatment effects [34]. Moreover, cluster‐randomized trials frequently involve varying cluster sizes (e.g., EDs with varying numbers of patients), which can further decrease statistical power [47]. As an extreme example, a trial comparing two clusters with 50 participants each would be more powerful than a trial comparing one cluster with 99 participants vs. another cluster with only one participant.

Second, cluster‐randomization can introduce both participant‐level and rater‐level biases in outcome ascertainment because it is difficult to blind both parties to the participant's randomization assignment when the entire clustering unit has been assigned to that condition [40]; this happens frequently when clusters receive their randomization assignment prior to individual participants being enrolled in the trial. Third, an inability to conceal allocation introduces the opportunity for recruitment or selection bias, particularly when individual patients are consented to study participation. This, in turn, has the potential to result in nontrivial imbalances in important baseline variables across comparator groups.

Finally, cluster‐randomization itself carries an increased risk of nontrivial imbalances in important baseline variables. In a cluster‐randomized trial, study arms should be comparable on baseline measures that pertain to individual patients as well as attributes of the clusters themselves [48, 49]; for example, a trial concerning trauma patients that randomizes medical centers should have study arms containing comparable patients (e.g., similar Revised Trauma Scores) and centers (e.g., similar number of Level I trauma centers). Though randomization can reduce the likelihood of imbalance if a large number of experimental units are randomized, cluster‐randomized trials typically randomize smaller numbers of experimental units (clusters) with variable patient populations and differing cluster sizes [4]. Indeed, it is not uncommon for cluster‐randomized trials to report imbalances on both cluster‐ and individual‐level variables at baseline.

## 5 Best Practices for Conducting Cluster‐Randomized Trials

When conducting or critically appraising a cluster‐randomized trial, it is important to be mindful of several best practices to maximize scientific validity. We note that there are existing guidelines and checklists that offer more detailed guidance on conducting cluster trials, if needed [40, 50, 51].

### 5.1 Justifying Cluster‐Randomization

First, trials should adequately justify their choice of cluster‐randomization over individual randomization with respect to the clinical question, target population, and intervention characteristics. This justification should begin with the assumption that an individually randomized trial design is preferred but, in this specific case, the advantages of cluster‐randomization outweigh the potential disadvantages. Additionally, if multiple levels of clustering exist (e.g., ICUs within a hospital, hospitals within a health system), the trial should justify its selection of the randomization unit. Reasons for selecting a higher order randomization unit over a lower order one tend to follow the same rationale for selecting cluster‐randomization over individual randomization: increased logistical convenience and a desire to avoid contamination, which then comes at the cost of decreased statistical efficiency and risk of chance imbalance.

### 5.2 Accounting for Clustered Data in Statistical Analysis

Second, a trial that randomizes by clusters must also account for the clustered nature of data in both the sample size calculation and statistical analysis plan. Sample size calculations for cluster‐randomized trials require an estimated intra‐cluster correlation (ICC) coefficient, in addition to the usual inputs for type I error rate (often denoted “alpha”), standard deviation, effect size, and power (1—“beta”). ICC values will vary depending on the clinical question and clustering unit, with higher values indicating a stronger within‐cluster correlation that necessitates enrolling a larger sample size. In general, increased sample size requirements can be addressed by increasing either the number of clusters or the size of each participating cluster. Depending on the ICC and the context of the trial, it is not uncommon to encounter scenarios where the power improves by increasing the number of clusters without increasing (or even decreasing) the total number of participants [52]. If cluster sizes are heterogeneous, which can also decrease power, sample size calculations should also account for this in some way. Finally, it is not uncommon for entire clusters to drop out of a trial (or never launch), and this should be considered during trial planning and sample size calculation. A number of existing software programs, such as the Shiny CRT Calculator [53], can be used to estimate sample size requirements across a range of cluster design types, cluster sizes, dropout rates, and ICC values (see Appendix S1: NEED‐PT Sample Size Table) [54]. In advanced trial designs, such as a stepped‐wedge rollout or bi‐directional crossover trial, researchers should consider how these differential design effects contribute to inflated sample size requirements [40].

Statistical methods for analyzing clustered data have been covered extensively in Wear et al.'s 2002 review [3], but in general proper analysis of clustered data either uses: (1) multilevel modeling (also called hierarchical, random‐effects, or mixed modeling), which explicitly estimates between‐cluster variation [29, 30, 55, 56], or (2) adjusting the standard errors and tests of more traditional linear models using cluster robust variance estimation [57]. In general, aggregating data by cluster (e.g., by averaging across observations within a cluster) is typically discouraged as it can generate misleading results when cluster sizes vary, a phenomenon known as ecological fallacy [40]. A technical appendix provides a brief introduction to the multilevel model approach (Appendix S2), and there are numerous examples of emergency care research studies using the multilevel modeling [23, 24, 25, 31] and cluster robust variance estimation [58] methods for analyzing clustered data.

### 5.3 Using Restricted Randomization

Third, researchers should consider using restricted randomization methods to decrease the risk of chance imbalance between comparator groups [49, 59, 60]. Methods such as covariate‐constrained randomization can incorporate cluster‐level covariates into the randomization process to exclude the most undesirable allocations while maintaining a high probability of random selection. In our recent trial of an embedded ED physical therapy care model for low back pain, we randomized 44 ED physicians to receive (or not receive) an ED physical therapist on their care team and incorporated physician characteristics such as gender, years in practice, number of fast‐track shifts, and opioid prescribing rate into a covariate‐constrained randomization method. Using mathematical combinatorics properties, we note that with 44 total physicians, there were over 2.1 trillion ways in which equal allocation of physicians across study arms could be achieved (i.e., there were over 2.1 trillion ways of selecting 22 physicians out of 44 for either arm). The constrained randomization technique simulated 10,000 possible random allocations, excluded a subset of random allocations that violated a pre‐specified threshold of allowable covariate imbalance, then randomly selected an allocation from the remaining “constrained” space [61], Other types of restricted randomization include stratification, matching, and minimization [49].

### 5.4 Justifying Waivers of Consent at All Levels of Research Participation

Finally, if the cluster‐randomized trial employs a waiver of informed consent, researchers should specify at which levels of research participation the waiver applied (randomization, intervention deliverer/target, inferential target) and provide an appropriate justification for excluding participants from the consent process with respect to (1) degree of risk, (2) logistical infeasibility of obtaining individual consent, and (3) potential adverse effects on rights and welfare. Such justification is necessary given the growing trend of cluster‐randomized trials involving a waiver of informed consent without appropriate justification [62, 63]. When a rationale is provided, cluster trials commonly cite the use of a group‐based intervention or a pragmatic research design as a reason for not obtaining consent. We note that although a pragmatic research design is often justified, the selection of a pragmatic trial design alone does not constitute a valid justification for waiving informed consent.

## 6 Special Ethical Considerations for Cluster‐Randomized Trials

Cluster trials can therefore bring about unique ethical considerations and challenges [64]. This complexity stems from the multiple levels of research participation involved in cluster trials: the clusters that are randomized, the clinicians within those clusters that deliver or receive the intervention, and the patients seen by the clinicians practicing at the study sites. Ideally, informed consent should be obtained at all levels; however, in practice, informed consent can be difficult to obtain at some levels for the same reason that cluster‐randomization was selected in the first place: logistical infeasibility. Take, for example, the Flexible In Duty Hour Requirements for Surgical Trainees (FIRST) trial, which randomized 117 U.S. general surgery residencies to either a standard or flexible duty‐hour policy waiving rules on maximum shift lengths and time off between shifts [20]. Residency program directors agreed to participate in the trial; however, individual residents were not consented to randomization as the intervention required all residents within a given program to be on the same schedule—speaking to the logistical infeasibility of both individual randomization and consent—and was considered to be minimal risk.

The FIRST trial met two of three requirements for waiving informed consent at the level of participating residents (logistical infeasibility, minimal risk); however, several commentators objected that the trial violated the rights and welfare of participating residents by subjecting them to increased fatigue, which in turn contributes to adverse outcomes at the resident and patient levels [65]. The FIRST trial investigators rebutted with well‐reasoned justifications for waiving informed consent and pointed out that the trial showed no difference in resident and patient outcomes; however, the ongoing debate highlights the dilemma that frequently arises in cluster trials due to the multiple levels of research participation—especially when one level involves a potentially vulnerable population who may be subject to undue influence and coercion. This is frequently the case when the unit of randomization is at the organizational level (e.g., hospital, physician practice) because the decision to participate in the trial is typically made by gatekeepers who may not necessarily represent the preferences of the organization's members yet hold positions of power over those members.

Even in cases where gatekeepers are not involved and randomized participants are consented to research, researchers should consider how the trial may unintentionally affect individuals downstream of the research participants and intervention (e.g., patients treated by participating clinicians) and seek to engage relevant patient stakeholders in trial planning. For a more detailed discussion of ethical considerations, we direct the reader towards the Ottawa Statement on the Ethical Design and Conduct of Cluster‐randomized Trials, which sets out 15 recommendations for the ethical design and conduct of cluster trials [66].

## 7 Concise Advice for Reading and Reviewing Cluster‐Randomized Trials

Finally, we provide the general emergency clinician reader with concise advice for reading and critically appraising published reports of cluster‐randomized trials. This list is not meant to be exhaustive, as there are more complete checklists for the accurate reporting of cluster‐randomized trials [50, 51, 66].

1. Identify the levels of research participants, with respect to who is being randomized, who is delivering and receiving the intervention, and who is providing the key information about outcomes, and thus the effect of the intervention.
2. Ask whether a cluster‐randomized design was appropriate for the research question, or conversely, whether an individual participant‐randomized design could have achieved the same objective.
3. Determine whether informed consent was obtained at each level of trial participation, and if not, whether waiving informed consent was justified.
4. Determine whether the clustered nature of data was accounted for in the sample size calculation and statistical analysis plan.
5. Evaluate whether clusters are different across the comparator groups, with respect to cluster sizes in the trial and pre‐trial cluster characteristics.
6. Evaluate whether individual participants in the comparator groups were dissimilar and if any differences affect your interpretation of the results.
7. Consider whether potential research participants were unaware of their allocation when considering participation, and if enrolled participants and researchers/outcome assessors were blinded to the randomization assignment, and if not, how this might have biased the results.

## 8 Conclusion

Cluster‐randomized trials are increasingly featured in emergency care research. Although cluster‐randomization can be a useful research approach, it must be appropriately matched to the right context with respect to the research question, target population, and intervention characteristics. Reasons for selecting a cluster‐randomized trial design, such as administrative or logistical convenience and the desire to reduce contamination, should be weighed against common limitations such as sample size inflation and possible research biases from lack of allocation concealment/blinding and chance imbalance.

## Author Contributions

H.S.K. contributed to the study concept and design, drafting of the manuscript, critical revision of the manuscript for important intellectual content, and acquisition of funding. J.M.S. and J.D.C. contributed to the study concept and design, drafting of the manuscript, critical revision of the manuscript for important intellectual content, and statistical expertise.

## Supporting information


**Appendix S1:** Example of a sample size calculation table from the NEED‐PT cluster‐randomized trial.

**Appendix S2:** Basics of analysis methods for cluster‐randomized trials.

## Data Availability

Data sharing not applicable to this article as no datasets were generated or analyzed during the current study.
