# Supplementary material for: Cluster‐Randomized Trials in Emergency Care Research
Source: Acad Emerg Med. 2025 Oct 28;33(2):e70181. doi: 10.1111/acem.70181 (PMC12875296; doi:10.1111/acem.70181)
Supplement: Supplementary file 1 — Appendix S1: Example of a sample size calculation table from the NEED‐PT cluster‐randomized trial. [file ACEM-33-0-s001.docx]

**Appendix A: Example of a Sample Size Calculation Table from the NEED-PT Cluster-Randomized Trial**

| **ICC** | **Physicians**  **(Total)** | **Physician % Dropout** | **Average N participants per Physician** | **Participant % Dropout** | **Power:**  **Mean 3.5pt Δ PROMIS-PI** |
| --- | --- | --- | --- | --- | --- |
| 0.01 | 40 | 0 | 9 | 0 | 97% |
|  | 40 | 0 | 8 | 5 to 10 | 95% |
|  | 40 | 0 | 7 | 15 to 20 | 92% |
|  | 38 | 5 | 9 | 0 | 96% |
|  | 38 | 5 | 8 | 5 to 10 | 94% |
|  | 38 | 5 | 7 | 15 to 20 | 90% |
|  | 36 | 10 | 9 | 0 | 95% |
|  | 36 | 10 | 8 | 5 to 10 | 92% |
|  | 36 | 10 | 7 | 15 to 20 | 89% |
|  | 34 | 15 | 9 | 0 | 94% |
|  | 34 | 15 | 8 | 5 to 10 | 91% |
|  | 34 | 15 | 7 | 15 to 20 | 87% |
|  | 32 | 20 | 9 | 0 | 92% |
|  | 32 | 20 | 8 | 5 to 10 | 89% |
|  | 32 | 20 | 7 | 15 to 20 | 85% |
| 0.05 | 40 | 0 | 9 | 0 | 96% |
|  | 40 | 0 | 8 | 5 to 10 | 94% |
|  | 40 | 0 | 7 | 15 to 20 | 91% |
|  | 38 | 5 | 9 | 0 | 95% |
|  | 38 | 5 | 8 | 5 to 10 | 93% |
|  | 38 | 5 | 7 | 15 to 20 | 90% |
|  | 36 | 10 | 9 | 0 | 94% |
|  | 36 | 10 | 8 | 5 to 10 | 91% |
|  | 36 | 10 | 7 | 15 to 20 | 88% |
|  | 34 | 15 | 9 | 0 | 93% |
|  | 34 | 15 | 8 | 5 to 10 | 90% |
|  | 34 | 15 | 7 | 15 to 20 | 86% |
|  | 32 | 20 | 9 | 0 | 91% |
|  | 32 | 20 | 8 | 5 to 10 | 88% |
|  | 32 | 20 | 7 | 15 to 20 | 84% |
| 0.10 | 40 | 0 | 9 | 0 | 96% |
|  | 40 | 0 | 8 | 5 to 10 | 94% |
|  | 40 | 0 | 7 | 15 to 20 | 91% |
|  | 38 | 5 | 9 | 0 | 95% |
|  | 38 | 5 | 8 | 5 to 10 | 93% |
|  | 38 | 5 | 7 | 15 to 20 | 90% |
|  | 36 | 10 | 9 | 0 | 94% |
|  | 36 | 10 | 8 | 5 to 10 | 92% |
|  | 36 | 10 | 7 | 15 to 20 | 88% |
|  | 34 | 15 | 9 | 0 | 93% |
|  | 34 | 15 | 8 | 5 to 10 | 90% |
|  | 34 | 15 | 7 | 15 to 20 | 86% |
|  | 32 | 20 | 9 | 0 | 91% |
|  | 32 | 20 | 8 | 5 to 10 | 88% |
|  | 32 | 20 | 7 | 15 to 20 | 84% |
