# Supplementary material for: Cluster‐Randomized Trials in Emergency Care Research
Source: Acad Emerg Med. 2025 Oct 28;33(2):e70181. doi: 10.1111/acem.70181 (PMC12875296; doi:10.1111/acem.70181)
Supplement: Supplementary file 2 — Appendix S2: Basics of analysis methods for cluster‐randomized trials. [file ACEM-33-0-s002.docx]

**Appendix B: Basics of Analysis Methods for Cluster-Randomized Trials**

This technical appendix provides a brief introduction of the methods typically used to analyze cluster-randomized trials. Though it provides some technical detail about options for model specification and estimation, it is hardly exhaustive. For additional information, see the works referenced in this section.

A naïve approach to analyzing clustered data, including cluster-randomized trials, involves aggregation of outcomes for individuals within clusters.^1^ For instance, in a trial that randomized emergency departments (EDs) to an intervention for which the primary outcome was a measure of patient quality of life, the method of aggregation would average the quality of life outcomes across patients in each ED and conduct an analysis of these averages, such as a t-test of ED averages comparing the treatment EDs to the control EDs.

Aggregation has several limitations. Conceptually, aggregating to the cluster level shifts the meaning of the outcome: it no longer pertains to individuals (e.g., patients), but the cluster (e.g., the ED).^1^ Aggregated analyses are subject to the ecological fallacy, which holds that relationships between cluster-level measures (even if they are averages of individual measures within clusters) are not necessarily indicative of relationships between measures on individuals.^2^ As an example, consider the comparison of rates of an adverse outcome across four EDs in two study arms. Suppose the control arm contains EDs A and B, both of which have a sample of 40 out 50 patients with the adverse outcome so that the mean for ED A is 80% and the mean for ED B is 80% (and hence the mean of their means is also 80%). In the treatment arm, suppose ED C has 90 patients, 54 of whom experience the adverse outcome, while ED D as 10 patients, all of whom experience the outcome. Thus, in the control arm, the mean for ED C is 60% and the mean of ED D is 100%, making the average of the two 80%. Yet, only 64/100 patients in the treatment arm experience the outcome.

Most statistical literature suggests that analyses of cluster-randomized trials should model the individual outcomes within clusters (e.g., patient outcomes within EDs) while accounting for the impact of clustering (e.g., adjusting for ED).^3,4^ The standard approach uses a linear mixed effects model (LMM), also referred to as a hierarchical or multilevel model. This approach can be adapted to adjust for baseline covariates and/or account for various outcome types (e.g., binary, count). Alternative approaches, including robust adjustments to standard errors and/or hypothesis tests are based on a similar, but more relaxed set of assumptions as LMMs and are used occasionally in analysis of cluster-randomized trials.

**A1. Basic Models for Cluster-Randomized Trials**

Consider a two-armed parallel cluster-randomized trial. For each cluster j, we would observe outcomes $Y_{ij}$ for person ${i=1, 2, \ldots, n}_{ij}$, as well as the treatment assignment $T_{j}$ such that $T_{j}$= 1 if cluster *j* was randomized to receive the treatment and $T_{j}$= 0 if cluster *j* was in randomized to receive the control condition.

A common model for estimating the effect of the treatment can be written as follows:

$Y_{ij}=\beta_{0}+\beta_{1}T_{j}+r_{j}+e_{ij}$ (1)

Here, $\beta_{0}$ would be interpreted as the mean of the control group, $\beta_{1}$ would be the effect of the treatment, $r_{j}$ is the random cluster effect and $e_{ij}$ is the random patient effect. This is referred to as a hierarchical linear model or a mixed effects model, as it includes effects assumed to be fixed ($\beta_{0}$ and $\beta_{1}$) and random ($r_{j}$). In its simplest form, it is typically assumed that the $r_{j}$ are idependent and identically distributed normal random variables with mean 0 and variance $\tau^{2}$. The $e_{ij}$are assumed to be independent of the $r_{j}$ and themselves be independent and identically distributed normal random variables with mean 0 and variance $\sigma^{2}$. In the statistical literature, $\tau^{2}$ is referred to the between-cluster variation, while $\sigma^{2}$ captures the within-cluster variation; together they are referred to as *variance components*. The total random variation in the outcomes $Y_{ij}$ is given by $\tau^{2}+\sigma^{2}$.

Readers familiar with standard (fixed effects) linear regression (occasionally called ordinary least squares or OLS) will find this equation familiar; the key difference from standard regression to model 1 above is the inclusion of the random cluster effect $r_{j}$. However, its inclusion has key implications for the model. It can be shown that given two outcomes in the same cluster $Y_{1j}$ and $Y_{2j}$ are correlated, and their correlation is given by $\tau^{2}/(\tau^{2}+ \sigma^{2})$, which is the expression for the ICC. While standard regression models assume that observations are independent, the inclusion of the random cluster effect inherently models a correlation between observations in the same cluster. Because observations are correlated, they contain less information about the population of interest than independent observations, and hence for the same total sample size, estimates of the fixed effects in model 1 ($\beta_{0}$ and $\beta_{1}$) tend to be less precise than if the $Y_{ij}$were independent. The relative decrease in precision can be quantified as a function of sample size (given the ICC), which is called the design effect.

Failing to account for within-cluster correlation can impact the accuracy of analyses. If the data follow model 1 above, but the cluster effect is ignored in the analysis of data (i.e., a standard regression model is used where a mixed effects model would be appropriate), the resulting analysis will underestimate standard errors of treatment effects and inflate test statistics and type I error rates.^5^

The parameters in equation (1), including the treatment effect and the variance components can be estimated using statistical techniques called maximum likelihood (ML) and restricted maximum likelihood (REML) estimation.^4^ The relevant distribution of test statistics, including the *t*-test for the treatment effect, are available in various treatments on this approach.^5-7^

**A2. Alternative Approaches**

While model 1 offers useful insight into a common model to analyze cluster-randomized trials, it is not the only approach. Model 1 can be expanded to include adjustments for individual- and cluster-level covariates analogous to an ANCOVA analysis in individually-randomized trials. Model 2 below includes a fixed-effect adjustment for a patient-level covariate $X_{ij}$ and a cluster-level covariate $W_{j}$:

$Y_{ij}=\beta_{0}+\beta_{1}T_{j}+\beta_{3}X_{ij}+ \beta_{4}W_{j}+r_{j}+e_{ij}$ (2)

Models 1-2 can also be modified to handle outcomes that are not continuous using a generalized linear mixed effects models (GLMM) framework. For instance, they could be rewritten as a mixed effects logistic regression model for binary outcomes or a mixed effects Poisson model for count outcomes.^4^

In addition, explicit assumptions regarding the distributions of the error terms $r_{j}$ and $e_{ij}$ can further be relaxed in the context of continuous outcomes. For instance, they need not be assumed to be normally distributed, an assumption that helps ensure *t*-tests referenced above have controlled Type I error rates. In the absence of this assumption, generalized estimating equations can be used to estimate the fixed effects, including the treatment effect $\beta_{1}$, while cluster-robust standard errors can appropriately adjust standard errors and hypothesis tests to ensure they are accurate.^8^ Moreover, methods there exists methods that adjust tests based on standard linear regression (i.e., regression without cluster effects) to better control Type I error rates.^5^

1. G. F. A Rule for Inferring Individual-Level Relationships from Aggregate Data. American Sociological Revew 1978;43(4):557-572.

2. Robinson WS. Ecological correlations and the behavior of individuals. Int J Epidemiol 2009;38(2):337-41. DOI: 10.1093/ije/dyn357.

3. Raudenbush SWB, A.S. Hierarchical Linear Models: Applications and Data Analysis Methods. 2nd ed: Sage, 2002.

4. Snijders TABB, R.J. Multilevel Analysis: An Introduction to Basic and Advanced Multilevel Modeling. 2nd ed: Sage, 2012.

5. Hedges LV. Correcting a significance test for clustering. Journal of Educational and Behavioral Statistics 2007;32(2):151-179. DOI: <https://doi.org/10.3102/1076998606298040>.

6. Manor O. ZDM. Small sample inference for the fixed effects in the mixed linear model. Comput Stat Data Anal 2004;46:801-817.

7. Raudenbush SW. Statistical analysis and optimal design for cluster randomized trials. Psychol Methods 1997;2(2):173-185. DOI: <https://doi.org/10.1037/1082-989X.2.2.173>.

8. Diggle P.J. HP, Liang K.Y., Zeger S.L. Analysis of Longitudinal Data. 2nd ed: Oxford University Press, 2002.
